# Predictors for County Level Variations in Initial 4-week COVID-19 Incidence and Case Fatality Risk in the United States

**DOI:** 10.21203/rs.3.rs-131858/v1

**Published:** 2020-12-21

**Authors:** Swapnil Khose, Hei Kit Chan, Henry E. Wang, Justin Xavier Moore

**Affiliations:** The University of Texas Health Science Center; The University of Texas Health Science Center at Houston; The University of Texas Health Science Center at Houston; Augusta University Medical Center

**Keywords:** case fatality risk, community characteristics, county level variation, COVID-19, incidence, predictors, obesity paradox

## Abstract

While studies indicate differences in incidence and case fatality risk of COVID-19, few efforts have shed light on regional variations in the intensity of initial community spread. We conducted a nationwide study using county-level data on COVID-19 from Center for Systems Science and Engineering at Johns Hopkins University. We characterized intensity of initial community COVID-19 attack by calculating the incidence and case fatality risk (CFR) for the first 4-week period of COVID-19 spread in each county. We used multivariate multilevel multinomial logistic regression to estimate the association of county-level characteristics with COVID-19 incidence and CFR. Of 3,143 counties, we included 1,052 with at least 100 reported cases on June 1st. Median incidence was 193.4 per 100,000 population (IQR: 94.2–397.5). Median case fatality risk was 3.6% (IQR: 1.4–7.3). Median age, rural population, population density, lower education, uninsured population, obesity, COPD prevalence were positively associated, while population, female sex, races (Asian, white), higher education, excessive drinking were negatively associated with initial COVID-19 incidence. Median age, female sex, Asian race, population density, higher education, excessive drinking, Intensive Care Unit beds, airborne infection isolation rooms were positively associated, while Hispanic ethnicity, lower education, obesity (paradox), uninsured population were negatively associated with initial COVID-19 CFR.

## Introduction

As of 10th August, 2020, the pandemic of COVID-19, caused by SARS-CoV-2, has claimed more than half a million lives worldwide. With nearly 5 million cases and 163,461 deaths, the United Sates has experienced the highest burden [1, 2]. While many studies have shown stark differences in incidence and case fatality risk of COVID-19 across different counties, some counties are being disproportionately affected than others [3–5]. Further, various studies have identified individual-level risk factors for COVID-19 hospitalization [6, 7], associated complications [8–13] as well as mortality [14–17]. However, there are very few studies examining the population-level factors explaining the differential rate of spread of COVID-19 infection as well as rate of fatality in different geographical regions [18–22].

Furthermore, few studies have focused on the initial community spread, which may indicate regions and communities particularly vulnerable to the effect of COVID-19. The initial intensity by which a disease spreads through a community may be influenced by numerous factors such as the virulence of the pathogen, the health behaviors of citizens, the biologic susceptibility of the population, or the health resources of the community. Understanding the factors responsible for the variation in initial incidence as well as case fatality risk could help efforts to identify high risk communities as well as targets for mitigating the spread of infection.

The primary objectives of this study were 1) To determine county level variations in initial COVID-19 incidence and case fatality risk indexed to the start of epidemic in each county and 2) To identify the predictors for county level variations in initial incidence and case fatality risk of COVID-19.

## Methods

### Study design and data source

We performed an ecological study examining the regional variation of COVID-19 across counties in the United States. We obtained county-level data on COVID-19 confirmed cases and deaths from the COVID-19 Data Repository by the Center for Systems Science and Engineering (CSSE) at Johns Hopkins University through 29th of June, 2020 [1, 2].

### Study population

We included counties with at least 100 cases on 1st June, 2020 to allow for 4-week period before we obtained the data i.e. 29th June, 2020.

### COVID-19 related outcomes

The primary outcomes of the study were incidence (number of new confirmed cases per 100,000 population) and case fatality risk [23] (CFR: ratio of number of new deaths and new confirmed cases, expressed as a percentage) of COVID-19. We calculated the incidence and case fatality risk for the 4-week period from the day of reporting at least 100 cases in each county to ensure fair comparison between counties. We focused primarily on initial community spread so as to identify high risk communities and their characteristics.

### Exposure (county-level community characteristics)

County-level data on socio-demographic factors, health behaviors, chronic medical conditions’ prevalence rates and availability of healthcare resources were obtained from the 2020 County Health Rankings (CHR) [24], 2018–2019 Area Health Resources File (AHRF) [25] and 2017 Centers for Medicare & Medicaid Services (CMS) [26] report on chronic medical conditions. We linked these county-level community characteristics with COVID-19 data using Federal Information Processing Standards (FIPS) codes. The details of sources and definitions for variables used can be found in the appendix I.

### Statistical analysis

We estimated descriptive statistics for COVID-19 outcomes as well as various community characteristics of the counties included in the study. We fit multilevel multinomial logistic regression models to estimate the association of county-level factors (socio-demographics, health behaviors, air pollution level, chronic medical conditions’ prevalence and availability of healthcare resources) with incidence and case fatality risk (CFR) of COVID-19. We used quartiles of incidence and CFR of COVID-19 as dependent variables. The models also constituted a random intercept for each state to account for unknown variations among states, such as weather, social distancing norms, timing of stay-at-home orders, etc. All models were adjusted for median age, sex (females) and race/ethnicity (Asian, Hispanic, non-Hispanic black, non-Hispanic white). All analyses were conducted at the county level. We performed all statistical analyses using STATA version 16.1 (Stata Corp, College Station, TX) and executed all mapping using ArcGIS version 10.4.

### IRB Statement

This study was considered exempt from Institutional Review Board (IRB) review as we used publicly available, population-level data.

## Results

**Figure 1:**
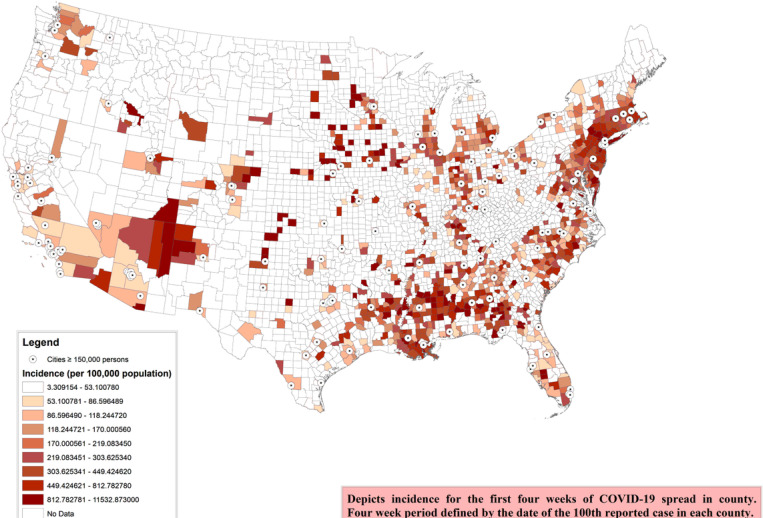
County-level 4-week COVID-19 incidence (per 100,000 population). Depicts incidence for the first four weeks of COVID-19 spread in each county. Four week period defined by the date of the 100th reported case in each county. Includes counties with at least 100 cases as of June 1 st, 2020 Note: The designations employed and the presentation of the material on this map do not imply the expression of any opinion whatsoever on the part of Research Square concerning the legal status of any country, territory, city or area or of its authorities, or concerning the delimitation of its frontiers or boundaries. This map has been provided by the authors.

**Figure 2:**
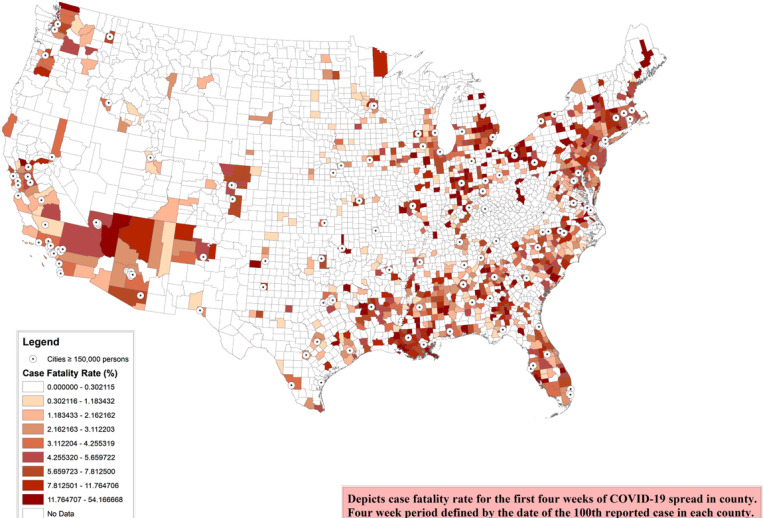
County-level 4-week COVID-19 case fatality risk Depicts case fatality risk for the rst four weeks of COVID-19 spread in each county. Four week period dened by the date of the 100th reported case in each county. Includes counties with at least 100 cases as of June 1st, 2020 Note: The designations employed and the presentation of the material on this map do not imply the expression of any opinion whatsoever on the part of Research Square concerning the legal status of any country, territory, city or area or of its authorities, or concerning the delimitation of its frontiers or boundaries. This map has been provided by the authors.

Of the total 3,143, we included 1,052 counties with at least 100 cases on 1st June, 2020. The characteristics of these counties are presented in Table 1. The median population among the counties was 105,100 with the inter quartile range (IQR) of 43,100 to 254,200. The mean ‘median age’ was 37.9 years with standard deviation (SD) of 4.2 years, while mean 16.9% (SD: 4.0) population were above 65 years of age. Mean 50.5% (SD: 2.0) of the population were females. The median incidence of COVID-19 among the counties included was 193.4 per 100,000 population (IQR: 94.2–397.5) (Fig. 1). The median case fatality risk (CFR) was 3.6% (IQR: 1.4–7.3) (Fig. 2).

We used multinomial regression to determine the association of county-level characteristics with the quartiles of 4- week COVID-19 incidence. Median age, rural population, population density, lower education (< HS Diploma), adult obesity prevalence, COPD prevalence, and uninsured population were positively associated with the highest quartile of incidence compared to the lowest quartile. While population, female sex, races (Asian and non-Hispanic white), higher education (HS diploma or more, 4 + years of college), and excessive drinking were negatively associated with the highest quartile of incidence. (Table 2)

Furthermore, we used multinomial regression to determine the association of county-level characteristics with the quartiles of 4- week case fatality risk (CFR) of COVID-19. Median age, female sex, Asian race, population density, higher education (HS diploma or more, 4 + years of college), excessive drinking, number of Intensive Care Unit (ICU) beds, and number of airborne infection isolation rooms were positively associated with the highest quartile of case fatality rates compared to the lowest quartile. Hispanic ethnicity, lower education (< HS diploma), adult obesity, uninsured population were negatively associated with the highest quartile of case fatality risk. (Table 3)

## Discussion

Ours is the first study to examine association of multiple population-level factors with the county-level variations in initial incidence and case fatality risk of COVID-19. We focused primarily on initial community spread so as to identify populations with higher susceptibility for COVID-19 infection and fatality. We found significant variation in the incidence (median: 193.4 per 100,000 population; inter quartile range (IQR): 94.2–397.5) as well as case fatality risk (CFR) of COVID-19 (median: 3.6%; IQR: 1.4–7.3) for the initial 4- week period.

We also identified various independent predictors of initial incidence of COVID-19. The positive association with higher median age, male sex, and chronic medical conditions (obesity and COPD) is in accordance with the various individual-level risk factors described by numerous clinical studies [6–10]. The elderly male populations with higher chronic disease burden are likely to have high susceptibility for COVID-19.

Interestingly, female sex was negatively associated with higher incidence. Biological susceptibility, occupational roles as well as responsible behavior with regard to following public health guidelines might explain this. Excessive drinking was also found to be strong protective factor, which could be explained by less mobility and social interaction by this population. On the other hand, population density was positively associated with higher incidence, supporting the role of social mobility in driving the spread of infection. All of these factors underscore the utility of social distancing in slowing the transmission of COVID-19. Additionally, higher education was negatively associated and percent uninsured population was positively associated with highest quartile of incidence. This highlights the importance of regular academic education as well as health education (percent uninsured population as proxy) in slowing the spread of the virus.

Furthermore, we identified independent predictors of case fatality risk of COVID-19 during initial community spread. Higher age and female sex were the strongest predictors associated with higher CFR, as shown by other individual-level clinical studies [14–17]. We also found significant positive association of Asian race with higher CFR, whereas Hispanic ethnicity was found to be negatively associated. Non-Hispanic black race was not found to be significantly associated with higher CFR. Various other studies have found non-significant association of black race with CFR [27–29], while some have shown significantly higher mortality [30]. Further research is needed in this area.

Unexpectedly, we did not find association of higher CFR with the prevalence of any of the included chronic medical conditions, except adult obesity. Adult obesity was negatively associated with the highest quartile of CFR (aOR: 0.95; 95% CI: 0.90, 0.99), supporting the ‘obesity paradox’. Obesity paradox has been described as an association of obesity with decrease in mortality in patients with acute respiratory distress syndrome (ARDS), reported previously in various studies [31–33]. However, whether such a phenomenon also holds true for ARDS following COVID-19 infection is not yet clear [32, 34].

Moreover, we found that fine particulate matter (PM 2.5) was not associated with CFR. This is in consonance with another nation-wide cross sectional study on effect of air pollution, which showed insignificant effect of PM 2.5 and Ozone, but significant effect of NO_2_ on COVID-19 death outcomes [22]. We also did not find independent association of smoking with CFR. However, different meta-analyses have identified significant associations of smoking with severe complications as well as higher mortality from COVID-19 [35, 36].

Surprisingly, availability of healthcare resource, defined by number of Intensive Care Unit beds and number of airborne infection isolation rooms, was found to be positively associated, although weakly, and uninsured population was found to be negatively associated with the highest quartile of case fatality risk. The lesser disease burden as well as rapidity of spread during the initial weeks of epidemic in each county might explain this contradictory effect of healthcare resources availability on CFR variation. A study in China showed that the rapid escalation in the number of infections around the epicenter of the outbreak (Wuhan city) resulted in an insufficiency of health-care resources, thereby negatively affecting mortality in Hubei province, but not in other provinces of China [37].

Our study included an assessment of comprehensive range of factors with potential predictability role for the spread and fatality of COVID-19. In contrast to other population-level studies on COVID-19, we were able to control for major confounding by epidemic timing as well as stage of the epidemic by identifying a common starting point for each county (i.e. reporting of first 100 cases). We also were able to control for the unmeasurable effect of various factors such as diverse weather, varied social distancing norms, different timing of stay-at-home orders, etc. by including the group effect for each state.

However, we do acknowledge that our study is limited in several key areas. Firstly, the data on confirmed cases and deaths of COVID-19 at CSSE at Johns Hopkins University is derived from publicly available data from multiple sources such as the World Health Organization, the U.S. Centers for Disease Control and Prevention, state and national government health departments, local media reports, etc [1, 2]. Because of the different COVID-19 case definitions used by different organizations, there could be an artificial variability in the data itself. Secondly, the case fatality risk estimation used does not provide the true rate, as there is a substantial lag of reported deaths among reported cases (most hospitalizations take 2–3 weeks till experiencing mortality) [38]. However, this is the limitation for all population-level studies. Thirdly, because of limited sample size, we were not able to control for all the plausible confounders in our modeling. Fourthly, we did not look at some other potential factors as it was beyond the scope of this study. Specifically, we could not examine the effect of important chronic medical conditions identified by various other studies, such as hypertension [39], chronic heart disease [40], cancer [41], etc. as well as other air pollutants such as NO_2_ & Ozone [22, 42, 43]. Fifthly, few chronic medical conditions’ data (asthma, COPD, chronic kidney disease) used in this study was obtained from CMS [26]. This is a Medicare beneficiary data and hence is not generalizable to the general population. Caution should be taken while interpreting the findings with respect to these three factors.

Since the beginning of the pandemic of novel coronavirus, there have been numerous efforts to build better prediction models. However, the predictability of these models has not been up to the expectation. The predictors identified by our study will definitely help build better models. Additionally, these findings may help identify most susceptible and high-risk populations and target public health interventions to focus areas. Lastly, our study also highlights the importance of social distancing as well as health education.

To summarize, we identified various county-level independent predictors of initial incidence as well as case fatality risk of COVID-19. The findings can help build better future prediction models. The results also support targeted public health actions by identifying susceptible and high-risk populations as well as counties.

## Supplementary Material

Supplement

## Figures and Tables

**Table 1:** Characteristics of counties (N = 1,052) included in study

Characteristics	Mean (standard deviation)
**COVID-19 Outcomes**	
4-week Incidence (per 100,000 population) *	193.4 (94.2– 397.5)
4-week Case Fatality Risk (CFR) (%) *	3.6 (1.4–7.3)
**Demographics**	
Population (thousands) *	105.1 (43.1–254.2)
Median Age (years)	37.9 (4.2)
65 and older (%)	16.9 (4.0)
Females (%)	50.5 (2.0)
Race (%)	
Non-Hispanic Black	15.2 (16.9)
Asian	2.8 (3.9)
Hispanic	11.5 (13.6)
Non-Hispanic White	67.6 (19.7)
Rural population (%)	35.0 (26.5)
Median household income (thousands of USD)	58.4 (17.2)
Unemployment rate (%)	4.1 (1.3)
Population Density (hundreds per square mile) *	1.7 (0.7–4.1)
Education (%)	
<HS Diploma	13.6 (6.1)
HS Diploma or more	86.4 (6.1)
4 + Years College	25.6 (11.1)
**Environmental**	
Air pollution - particulate matter (μg/m^3^)	9.9 (1.7)
**Health Behaviors** (%)	
Adult smoking	17.1 (3.3)
Excessive drinking	17.9 (3.1)
**Chronic medical Conditions prevalence (%)**	
Diabetes	11.8 (3.6)
Adult obesity	32.5 (5.7)
HIV (per 100,000 population) *	186.7 (111.1–320.2)
Asthma ^#^	5.0 (1.0)
Chronic Kidney Disease ^#^	24.4 (3.8)
COPD ^#^	12.3 (3.1)
**Healthcare Resources**	
Population to primary care physician ratio *	1,710 (1,209–2,424)
Uninsured population (%)	12.9 (6.0)
Number of Hospital Beds available *	244 (69–770)
Number of ICU Beds available *	11 (0–38)
Number of Airborne Infection Isolation Rooms available *	8 (1–33)

Abbreviations: COPD- Chronic Obstructive Pulmonary Disease; HS- High School; HIV- Human Immunodeficiency Virus; ICU- Intensive Care Unit; USD- United Sates Dollars.

* median (inter quartile range)

# among Medicare beneficiaries

**Table 2:** Association of county-level characteristics with the quartiles of 4- week COVID-19 incidence (1st quartile as a reference category). Table depicts result of multilevel multinomial logistic regression. All models were adjusted for median age, sex (females) and race/ethnicity (Asian, Hispanic, non-Hispanic black, non-Hispanic white).

Factors	Incidence					
	2nd Quartile		3rd Quartile		4th Quartile	
	aOR	95% CI	aOR	95% CI	aOR	95% CI
**Demographics**						
Population (thousands)	1.00	0.99, 1.00	0.99	0.99, 1.00	0.99	0.99, 0.99
Median Age (years)	1.05	0.99, 1.12	1.06	1.00, 1.12	1.14	1.07, 1.21
65 and older (%)	0.90	0.80, 1.01	0.92	0.82, 1.04	0.98	0.88, 1.09
Females (%)	0.75	0.63, 0.90	0.75	0.63, 0.90	0.62	0.52, 0.73
Race (%)						
Non-Hispanic Black	1.07	0.99, 1.17	1.02	0.97, 1.07	1.02	0.97, 1.07
Asian	1.01	0.91, 1.12	0.91	0.84, 0.99	0.92	0.85, 0.99
Hispanic	1.05	0.97, 1.14	1.00	0.95, 1.04	1.00	0.96, 1.04
Non-Hispanic White	1.02	0.94, 1.11	0.96	0.92, 1.01	0.94	0.90, 0.98
Rural population (%)	1.01	1.00, 1.02	1.02	1.01, 1.03	1.03	1.02, 1.04
Median household income (thousands of USD)	1.01	1.00, 1.03	1.00	0.98, 1.02	0.99	0.98, 1.01
Unemployment rate (%)	0.88	0.71, 1.09	0.98	0.80, 1.20	0.92	0.76, 1.11
Population Density (hundreds per square mile)	1.01	0.99, 1.04	1.02	0.99, 1.04	1.03	1.01, 1.05
Education (%)						
<HS Diploma	1.08	1.01, 1.14	1.17	1.11, 1.24	1.25	1.18, 1.32
HS Diploma or more	0.93	0.87, 0.99	0.85	0.80, 0.90	0.80	0.76, 0.85
4 + Years College	0.99	0.97, 1.02	0.97	0.95, 1.00	0.95	0.92, 0.97
**Environmental**						
Air pollution - particulate matter 2.5 (μg/m^3^)	1.19	1.02, 1.40	1.18	1.00, 1.38	0.97	0.82, 1.14
**Health Behavior** (%)						
Adult smoking	0.99	0.89, 1.09	1.04	0.94, 1.15	1.09	0.98, 1.20
Excessive drinking	0.89	0.81, 0.99	0.90	0.81, 0.99	0.86	0.78, 0.96
**Chronic medical Conditions prevalence** (%)						
Diabetes	0.97	0.90, 1.05	1.06	0.98, 1.14	1.07	1.00, 1.16
Adult obesity	1.02	0.98, 1.07	1.04	0.99, 1.09	1.05	1.01, 1.11
HIV (per 100,000 population)	1.00	0.99, 1.00	1.00	0.99, 1.00	1.00	0.99, 1.00
Asthma ^#^	0.96	0.77, 1.19	0.97	0.78, 1.21	0.82	0.65, 1.02
Chronic Kidney Disease ^#^	1.03	0.97, 1.10	1.02	0.96, 1.09	1.00	0.94, 1.06
COPD ^#^	1.05	0.97, 1.14	1.09	1.01, 1.18	1.10	1.01, 1.20
**Healthcare Resources**						
Population to primary care physician ratio	1.00	0.99, 1.00	1.00	0.99, 1.00	1.00	0.99, 1.00
Uninsured (%)	1.09	1.01, 1.17	1.09	1.01, 1.17	1.09	1.02, 1.18
Hospital Beds	1.00	0.99, 1.00	1.00	0.99, 1.00	1.00	0.99, 1.00
ICU Beds	1.00	0.99, 1.00	1.00	0.99, 1.00	0.99	0.99, 0.99
Airborne Infection Isolation Rooms	1.00	0.99, 1.00	1.00	0.99, 1.00	1.00	0.99, 1.00

Abbreviations: aOR- adjusted Odds Ratio; COPD- Chronic Obstructive Pulmonary Disease; CI – Confidence Interval; HS- High School; HIV- Human Immunodeficiency Virus; ICU- Intensive Care Unit; USD- United Sates Dollars.

# among Medicare beneficiaries.

**Table 3:** Association of county-level characteristics with quartiles of 4- week COVID-19 case fatality risk (1st quartile as a reference category). Table depicts result of multilevel multinomial logistic regression. All models were adjusted for median age, sex (females) and race/ethnicity (Asian, Hispanic, non-Hispanic black, non-Hispanic white).

Factors	Case Fatality Risk (CFR)					
	2nd Quartile		3rd Quartile		4th Quartile	
	aOR	95% CI	aOR	95% CI	aOR	95% CI
**Demographics**						
Population (thousands)	1.00	1.00, 1.01	1.00	1.00, 1.00	1.00	1.00, 1.01
Median Age (years)	1.09	1.03, 1.16	1.13	1.06, 1.20	1.16	1.09, 1.23
65 and older (%)	0.92	0.82, 1.04	0.92	0.81, 1.03	0.97	0.86, 1.09
Females (%)	1.25	1.12, 1.38	1.55	1.34, 1.79	1.26	1.13, 1.40
Race (%)						
Non-Hispanic Black	1.00	0.96, 1.04	1.02	0.97, 1.07	1.01	0.96, 1.06
Asian	1.23	1.12, 1.36	1.28	1.15, 1.42	1.15	1.03, 1.29
Hispanic	0.99	0.95, 1.03	1.00	0.95, 1.05	0.94	0.90, 0.99
Non-Hispanic White	0.97	0.93, 1.01	0.99	0.94, 1.04	0.98	0.94, 1.03
Rural population (%)	0.99	0.98, 1.00	0.98	0.97, 0.99	0.99	0.98, 1.00
Median household income (thousands of USD)	1.02	1.00, 1.04	1.03	1.01, 1.05	1.01	0.99, 1.03
Unemployment rate (%)	0.86	0.70, 1.05	0.93	0.76, 1.14	1.22	1.00, 1.48
Population Density (hundreds per square mile)	1.05	1.01, 1.09	1.05	1.01, 1.10	1.05	1.01, 1.10
Education (%)						
<HS Diploma	0.93	0.89, 0.98	0.91	0.86, 0.95	0.95	0.90, 0.99
HS Diploma or more	1.07	1.02, 1.12	1.10	1.05, 1.16	1.06	1.01, 1.11
4 + Years College	1.04	1.02, 1.07	1.05	1.03, 1.08	1.03	1.01, 1.06
**Environmental**						
Air pollution - particulate matter 2.5 (μg/m^3^)	1.11	0.96, 1.28	1.14	0.98, 1.33	1.09	0.93, 1.27
**Health Behavior** (%)						
Adult smoking	0.88	0.80, 0.98	0.86	0.77, 0.95	0.91	0.82, 1.00
Excessive drinking	1.14	1.04, 1.26	1.20	1.08, 1.32	1.14	1.03, 1.26
**Chronic medical Conditions prevalence** (%)						
Diabetes	0.99	0.93, 1.06	0.92	0.86, 0.99	0.95	0.88, 1.02
Adult obesity	0.96	0.92, 1.00	0.95	0.90, 0.99	0.95	0.90, 0.99
HIV (per 100,000 population)	1.00	1.00, 1.00	1.00	1.00, 1.00	1.00	1.00, 1.00
Asthma ^#^	0.90	0.73, 1.10	1.06	0.86, 1.30	0.91	0.74, 1.12
Chronic Kidney Disease ^#^	1.03	0.97, 1.10	1.05	0.98, 1.11	1.03	0.97, 1.10
COPD ^#^	0.94	0.87, 1.01	0.95	0.88, 1.03	0.98	0.91, 1.06
**Healthcare Resources**						
Population to primary care physician ratio	1.00	0.99, 1.00	1.00	0.99, 1.00	1.00	0.99, 1.00
Uninsured (%)	0.95	0.90, 1.01	0.90	0.84, 0.95	0.93	0.88, 0.99
Hospital Beds	1.00	1.00, 1.00	1.00	1.00, 1.00	1.00	1.00, 1.00
ICU Beds	1.02	1.01, 1.03	1.02	1.01, 1.03	1.02	1.01, 1.03
Airborne Infection Isolation Rooms	1.02	1.01, 1.03	1.02	1.01, 1.03	1.02	1.01, 1.02

Abbreviations: aOR- adjusted Odds Ratio; COPD- Chronic Obstructive Pulmonary Disease; CI – Confidence Interval; HS- High School; HIV- Human Immunodeficiency Virus; ICU- Intensive Care Unit; USD- United Sates Dollars.

# among Medicare beneficiaries.
