# Supplementary material for: Predictors for County Level Variations in Initial 4-week COVID-19 Incidence and Case Fatality Risk in the United States
Source: Res Sq. 2020 Dec 21:rs.3.rs-131858. Preprint. [Version 1] doi: 10.21203/rs.3.rs-131858/v1 (PMC7781326; doi:10.21203/rs.3.rs-131858/v1)
Supplement: Supplement [file 2b8e3cd0eec5c2d0c48537aa.docx]

**Appendix A**

**Description of variables used in the study**

| **Measure** | **Description** | **Data File Source** | **Source** | **Year** |
| --- | --- | --- | --- | --- |
| **Demographics** | | | | |
| Population | Resident population. | CHR | Census Population Estimates | 2018 |
| Median Age | The age where half of population in a county age more and half of population age less. | HRSA | Area Health Resources File (AHRF) 2018-2019 | 2010 |
| 65 and older | Percentage of population ages 65 and older. | CHR | Census Population Estimates | 2018 |
| Females | Percentage of population that is female. | CHR | Census Population Estimates | 2018 |
| Non-Hispanic Black | Percentage of population that is non-Hispanic Black or African American. | CHR | Census Population Estimates | 2018 |
| Asian | Percentage of population that is Asian. | CHR | Census Population Estimates | 2018 |
| Hispanic | Percentage of population that is Hispanic. | CHR | Census Population Estimates | 2018 |
| Non-Hispanic White | Percentage of population that is non-Hispanic White. | CHR | Census Population Estimates | 2018 |
| <HS Diploma | Percentage of Persons 25+ w/<HS Diploma | HRSA | Area Health Resources File (AHRF) 2018-2019 | 2013-17 |
| HS Diploma or more | Percentage of Persons 25+ w/HS Diploma or more | HRSA | Area Health Resources File (AHRF) 2018-2019 | 2013-17 |
| 4+ Years College | Percentage of Persons 25+ w/4+ Years College | HRSA | Area Health Resources File (AHRF) 2018-2019 | 2013-17 |
| Rural population | Percentage of population living in a rural area. | CHR | Census Population Estimates | 2010 |
| Median household income | The income where half of households in a county earn more and half of households earn less. | CHR | Small Area Income and Poverty Estimates | 2018 |
| Unemployment rate | Percentage of population ages 16 and older unemployed but seeking work. | CHR | Bureau of Labor Statistics | 2018 |
| Population Density | Population Density per Square Mile | HRSA | Area Health Resources File (AHRF) 2018-2019 | 2010 |
| **Environmental** | | | | |
| Air pollution - particulate matter raw value | Average daily density of fine particulate matter in micrograms per cubic meter (PM2.5). | CHR | Environmental Public Health Tracking Network | 2014 |
| **Health Behaviors** | | | | |
| Adult smoking | Percentage of adults who are current smokers. | CHR | Behavioral Risk Factor Surveillance System | 2017 |
| Excessive drinking | Percentage of adults reporting binge or heavy drinking. | CHR | Behavioral Risk Factor Surveillance System | 2017 |
| **Chronic medical conditions prevalence** | | | | |
| Diabetes prevalence | Percentage of adults aged 20 and above with diagnosed diabetes. | CHR | United States Diabetes Surveillance System | 2016 |
| Adult obesity | Percentage of the adult population (age 20 and older) that reports a body mass index (BMI) greater than or equal to 30 kg/m2. | CHR | United States Diabetes Surveillance System | 2016 |
| HIV prevalence | Number of people aged 13 years and older living with a diagnosis of human immunodeficiency virus (HIV) infection per 100,000 population. | CHR | National Center for HIV/AIDS, Viral Hepatitis, STD, and TB Prevention | 2016 |
| Asthma | Number of beneficiaries with asthma divided by the total number of beneficiaries in CMS fee-for-service population, expressed as a percentage | CMS | CMS Chronic Condition Data Warehouse | 2017 |
| COPD | Number of beneficiaries with COPD divided by the total number of beneficiaries in CMS fee-for-service population, expressed as a percentage | CMS | CMS Chronic Condition Data Warehouse | 2017 |
| Chronic kidney disease | Number of beneficiaries with ‘Chronic kidney disease’ divided by the total number of beneficiaries in CMS fee-for-service population, expressed as a percentage | CMS | CMS Chronic Condition Data Warehouse | 2017 |
| **Healthcare Resources** | | | | |
| Uninsured | Percentage of 18-64 year old without Health Insurance | HRSA | Area Health Resources File (AHRF) 2018-2019 | 2017 |
| Primary care physicians | Number of primary care physicians per 100,000 population | HRSA/ CHR | Area Health Resource File/American Medical Association | 2017 |
| Population to primary care physicians ratio | Ratio of population to primary care physicians | HRSA/ CHR | Area Health Resource File/American Medical Association | 2017 |
| Hospital Beds | Number of Hospital Beds available | HRSA | Area Health Resources File (AHRF) 2018-2019 | 2017 |
| ICU beds | Number of Med/Surg Intensive Care Unit beds available | HRSA | Area Health Resources File (AHRF) 2018-2019 | 2017 |
| Airborne Infection Isolation Rooms | Number of Airborne Infection Isolation Rooms available | HRSA | Area Health Resources File (AHRF) 2018-2019 | 2017 |
| **Abbreviations:** CHR- County Health Rankings; CMS - Centers for Medicare & Medicaid Services; COPD- Chronic Obstructive Pulmonary Disease; HIV- Human Immunodeficiency Virus; HRSA - Health Resources and Services Administration; HS- High School; ICU- Intensive Care Unit; USD- United Sates Dollars | | | | |

**Appendix B**

List of top 20 counties with highest 4-week Incidence of COVID-19

| **Rank** | **County name** | **State** | **Date of reporting first 100 cases** | **Number of new cases in**  **4-week period** | **Population** | **Incidence**  **(per 100,000 population)** |
| --- | --- | --- | --- | --- | --- | --- |
|  |  |  |  |  |  |  |
| 1 | Trousdale | Tennessee | 29-Apr-20 | 1270 | 11,012 | 11532.87 |
| 2 | Buena Vista | Iowa | 17-May-20 | 1486 | 19,874 | 7477.10 |
| 3 | Lincoln | Arkansas | 18-Apr-20 | 845 | 13,383 | 6313.98 |
| 4 | Dakota | Nebraska | 23-Apr-20 | 1243 | 20,083 | 6189.31 |
| 5 | Nobles | Minnesota | 21-Apr-20 | 1293 | 21,924 | 5897.64 |
| 6 | Lake | Tennessee | 13-May-20 | 430 | 7,411 | 5802.18 |
| 7 | New York | New York | 13-Mar-20 | 92230 | 1,628,701 | 5662.79 |
| 8 | Colfax | Nebraska | 1-May-20 | 496 | 10,881 | 4558.40 |
| 9 | Cass | Indiana | 21-Apr-20 | 1453 | 37,955 | 3828.21 |
| 10 | Texas | Oklahoma | 28-Apr-20 | 783 | 20,455 | 3827.91 |
| 11 | Ford | Kansas | 18-Apr-20 | 1204 | 33,888 | 3552.88 |
| 12 | Marion | Ohio | 14-Apr-20 | 2302 | 65,256 | 3527.64 |
| 13 | Finney | Kansas | 23-Apr-20 | 1271 | 36,611 | 3471.63 |
| 14 | East Carroll | Louisiana | 25-May-20 | 236 | 7,037 | 3353.70 |
| 15 | Pickaway | Ohio | 15-Apr-20 | 1786 | 58,086 | 3074.75 |
| 16 | Seward | Kansas | 22-Apr-20 | 668 | 21,780 | 3067.03 |
| 17 | Sevier | Arkansas | 26-May-20 | 495 | 17,139 | 2888.15 |
| 18 | Dawson | Nebraska | 19-Apr-20 | 681 | 23,709 | 2872.32 |
| 19 | Rockland | New York | 20-Mar-20 | 8886 | 325,695 | 2728.31 |
| 20 | Chattahoochee | Georgia | 1-Jun-20 | 291 | 10,684 | 2723.69 |

**Appendix C**

List of top 20 counties with highest case fatality risk (CFR) of COVID-19

| **Rank** | **County name** | **State** | **Number of deaths** | **Number of cases** | **CFR (%)** |
| --- | --- | --- | --- | --- | --- |
|  |  |  |  |  |  |
| 1 | Orange | Indiana | 13 | 24 | 54.16 |
| 2 | Jackson | West Virginia | 16 | 36 | 44.44 |
| 3 | St. Landry | Louisiana | 43 | 101 | 42.57 |
| 4 | Washington | Ohio | 7 | 18 | 38.88 |
| 5 | Miami | Ohio | 15 | 43 | 34.88 |
| 6 | Wagoner | Oklahoma | 10 | 29 | 34.48 |
| 7 | McMinn | Tennessee | 13 | 41 | 31.70 |
| 8 | Ottawa | Ohio | 7 | 24 | 29.16 |
| 9 | Steuben | New York | 31 | 108 | 28.70 |
| 10 | Lapeer | Michigan | 23 | 84 | 27.38 |
| 11 | Tioga | New York | 8 | 30 | 26.66 |
| 12 | Hancock | Georgia | 23 | 87 | 26.43 |
| 13 | Oconee | Georgia | 7 | 28 | 25.00 |
| 14 | Randolph | Georgia | 16 | 65 | 24.61 |
| 15 | Greene | Indiana | 17 | 70 | 24.28 |
| 16 | Boone | Indiana | 28 | 116 | 24.13 |
| 17 | Grant | Indiana | 16 | 67 | 23.88 |
| 18 | Yolo | California | 18 | 76 | 23.68 |
| 19 | Otsego | Michigan | 4 | 17 | 23.52 |
| 20 | Baton Rouge | Louisiana | 12 | 51 | 23.52 |
